# Serum VEGF Potential as a Biomarker for Diagnosis of Female Unexplained Infertility: Does Atopy Matter?

**DOI:** 10.3390/ijms26199499

**Published:** 2025-09-28

**Authors:** Gabija Didžiokaitė, Austėja Grudytė, Aida Kuznecovaitė, Margarita Žvirblė, Žilvinas Survila, Vita Pašukonienė, Violeta Kvedarienė

**Affiliations:** 1 Faculty of Medicine, Vilnius University, LT-01513 Vilnius, Lithuania; 2 Department of Pathology, Institute of Biomedical Science, Faculty of Medicine, Vilnius University, LT-01513 Vilnius, Lithuania; 3 National Cancer Institute, P. Baublio Str. 3B, LT-08406 Vilnius, Lithuania; 4 Institute of Biosciences, Life Sciences Center, Vilnius University, Saulėtekio Av. 7, LT-10257 Vilnius, Lithuania; 5 Clinic of Chest Diseases, Immunology and Allergology, Institute of Clinical Medicine, Faculty of Medicine, Vilnius University, LT-01513 Vilnius, Lithuania; 6 Center of Innovative Allergology, M. Mažvydo Str. 13-47, LT-06256 Vilnius, Lithuania

**Keywords:** unexplained infertility, female reproductive health, vascular endothelial growth factor (VEGF), biomarker, allergic sensitization, atopy, ALEX2 macroarray

## Abstract

Unexplained infertility (UI) remains a diagnostic challenge affecting a significant proportion of women of reproductive age. Vascular endothelial growth factor (VEGF), a key mediator of angiogenesis and inflammation, has been implicated in reproductive and immune processes. This prospective observational study evaluated serum VEGF levels and allergen sensitization (via ALEX^2^ macroarray) in 70 women—51 with UI and 19 fertile controls—to assess VEGF’s potential as a biomarker in UI. Median VEGF concentrations were higher in women with UI compared to fertile controls (128.6 pg/mL vs. 82.5 pg/mL), though not statistically significantly. However, sensitized women showed significantly elevated VEGF levels compared to non-sensitized peers (115.9 pg/mL vs. 85.7 pg/mL, *p* = 0.028), and a stepwise increase in VEGF was observed with rising allergy severity (*p* = 0.045). Sensitization to pet allergens, particularly cat allergen Fel d 1, was associated with the highest VEGF levels. A literature review confirmed wide variability in VEGF concentrations and the lack of standardized norms. While VEGF alone may not serve as a definitive biomarker for infertility, elevated levels may reflect an underlying inflammatory state. Our findings suggest VEGF testing could support broader clinical evaluation in women with UI, especially in the presence of allergic sensitization.

## 1. Introduction

Infertility is a growing issue, affecting between 12.6% and 17.5% of couples of reproductive age worldwide [1]. In around 15% of cases, the cause of infertility remains unknown even after a thorough investigation of both partners [2]. In these cases, “infertility of unknown origin” or “unexplained infertility” (UI) is diagnosed. Currently, there is no standardized management algorithm for couples with UI in order to take targeted actions to improve their fertility outcomes. This lack of standardized management highlights a critical gap in understanding the pathophysiology of UI and identifying reliable diagnostic or prognostic markers.

Recent studies have investigated various biomarkers to uncover the underlying causes and mechanisms that could be the real underlying reason of infertility for patients, diagnosed with unexplained infertility [3,4]. Among them is vascular endothelial growth factor (VEGF), which is a crucial mediator of endothelial cell proliferation, differentiation, and survival, along with influencing vascular permeability [5]. The existing data suggests that VEGF in woman’s body also serves in embryo implantation, and changes in its expression, such as VEGF polymorphisms, might contribute to both infertility and pregnancy-related complications [6]. Therefore, comprehensive analysis of VEGF as a potential biomarker in unexplained infertility and related reproductive disorders for women could provide valuable insights into the underlying pathophysiological mechanisms of cases previously classified as idiopathic.

VEGF is also an angiogenic factor and a protein, essential for vascular function, tissue repair, and embryonic development. This protein has an influence for pathologies ranging from cancer to chronic inflammatory diseases like asthma [5,7,8]. In the female reproductive system, VEGF plays a crucial role during pregnancy in vasculogenesis, the formation of the embryonic circulatory system, as well as angiogenesis—the development of blood vessels from existing vasculature. Signal transduction occurs when VEGF binds to tyrosine kinase receptors, triggering a cascade that leads to endothelial cell proliferation, migration, and the formation of new blood vessels [7,8,9]. Physiological angiogenesis primarily occurs in wound healing and during the female reproductive cycle [7]. In this context, the investigation of VEGF as a biomarker in reproductive health is essential, especially given the complex and multifactorial nature of female infertility. As VEGF is regulated by several factors—including atopy, which has also been implicated in infertility—it needs to be carefully considered in clinical assessments of reproductive well-being [10].

Sensitization, which is a heightened immune response to environmental allergens, has been linked to unexplained infertility through its impact on the immune system and uterine environment. Increased levels of inflammatory markers, such as IgE and cytokines like IL-6 and TNF-α, can disrupt endometrial receptivity, making implantation more difficult [11]. Chronic low-grade inflammation, driven by allergens, may further alter vascular factors like VEGF, which are crucial for healthy blood vessel formation in the uterus [12]. The absence of clear reference ranges and the multifactorial regulation of VEGF further complicate its interpretation in clinical practice. Therefore, the aim of our study was to analyze VEGF’s potential as a biomarker for unexplained infertility for women, taking into consideration its multifaceted nature and challenging interpretation, and to evaluate the possible connections between VEGF concentrations, infertility and atopic sensitization in women. Additionally, in order to establish the range of “normal” serum VEGF concentrations for healthy individuals and to facilitate comparison with the data obtained in our study, we have performed a literature review of reported serum VEGF value ranges in scientific literature published between 1998 and 2024. Addressing these gaps is essential to determine whether VEGF could serve as a meaningful biomarker in UI and guide more personalized reproductive care strategies.

## 2. Results

The total of 70 women were included in the study: 51 women with primary unexplained infertility (UI) and 19 fertile women. A total of 6 women with UI did not agree to disclose their personal data; therefore, only their serum VEGF concentrations and ALEX^2^ macroarray test’s results were included in this study, while analysis of demographical data and other infertility related factors was only performed for 45 women with primary UI. The average age of the subjects was 33.58 (SD ± 5) years with a minimum of 18 and a maximum of 43 years old. The average age of fertile women was 32.89 (SD ± 6.71) and the average age of women with UI—33.87 (SD ± 4.19). Of 64 women included in this study with known anamnesis, 35 had additional chronic, allergic or autoimmune diseases, of which 7 were fertile and 28 were infertile, while only 29 women did not have any comorbidities. Infertile women were diagnosed with autoimmune diseases statistically significantly more frequently in comparison with fertile women (10 vs. 0 (*p* = 0.026)).

6 subjects from the fertile group and 18 from the infertile group were diagnosed with allergic diseases. The most common allergic diseases in UI group were allergic rhinitis (9 (20.00%) and atopic dermatitis (9 (20.00%), in fertile women group—atopic dermatitis (5 (26.32%)). There was no statistically significant difference between the prevalence of allergic diseases in general between fertile and infertile women in the sample of our study (*p* = 0.583) as well as between the prevalence of specific allergic diseases, such as allergic rhinitis, atopic dermatitis, urticaria, bronchial asthma, etc. 

In order to determine the participants allergy status, ALEX^2^ macroarray tests were performed for all participants. Allergy was confirmed if a correlation between hypersensitization and clinical symptoms for a specific allergen was confirmed. If no clinical history of allergy was determined for a subject, while ALEX^2^ indicated a sensitization, atopy was diagnosed. The associations between allergy and atopy status, ALEX^2^ results and serum VEGF concentrations were analyzed.

A total of 37 subjects were diagnosed with sensitization to at least one allergen component. Among these, 20 had confirmed allergies as determined by the ALEX^2^ macroarray test and showed clinical manifestations. Meanwhile, 16 subjects were diagnosed with sensitization but either did not exhibit clinical symptoms or lacked sufficient clinical data for interpretation; these individuals were categorized into the atopic patient group. There were no statistically significant differences between the prevalence of allergy and atopy between fertile and infertile women; however, women with UI were diagnosed with confirmed allergy slightly more frequently in comparison with fertile women: 15 (33.33%) infertile vs. 5 (26.32%) fertile (*p* = 0.769). The median VEGF blood serum concentration of the whole sample group was 111.6 pg/mL (IQR = 134.2). The median serum VEGF concentration for women, diagnosed with infertility of unknown origin, was higher in comparison with fertile women, 128.6 pg/mL vs. 82.5 pg/mL, *p* = 0.152. Serum VEGF levels in women with unexplained infertility ranged from 0.0 pg/mL to 501.7 pg/mL, while levels in fertile women ranged from 5.1 pg/mL to 443.9 pg/mL. Also, VEGF values of different allergy status subgroups were compared (Table 1).

**Figure 1:**
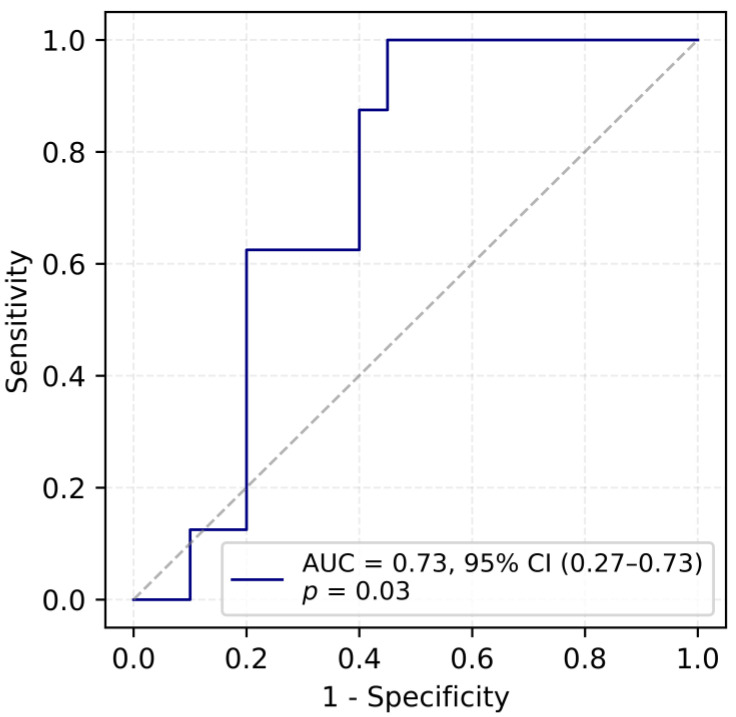
ROC curve for predicting fertility outcomes based on VEGF concentration.

When the data was introduced into the logistic regression model, it was indicated that serum VEGF level exhibits a moderate ability to discriminate between fertile and infertile women with an area under the ROC curve (AUC) of 0.73. The optimal cut-off value, determined using Youden’s J statistic, was identified as 122.23 pg/mL, corresponding to a sensitivity of 100% and a specificity of 55%. These findings suggest that serum VEGF concentration may have utility as a classifier for fertility status, given its overall ability to distinguish between fertile and infertile women (Figure 1).

Median serum VEGF values were compared between sensitized women, according to ALEX^2^ macroarray test and non-sensitized women considering whether sensitization may also determine higher levels of serum VEGF concentration. Firstly, it was observed that the median serum VEGF concentration of women who were sensitized to at least one allergen was statistically significantly higher in comparison with non-sensitized women (115.9 pg/mL vs. 85.7 pg/mL, accordingly), *p* = 0.028. Secondly, when fertile and infertile women were compared according to their sensitization status, it was determined that in both fertile and infertile groups, serum VEGF concentrations were higher in the sensitized group in comparison with non-sensitized group.

**Figure 2:**
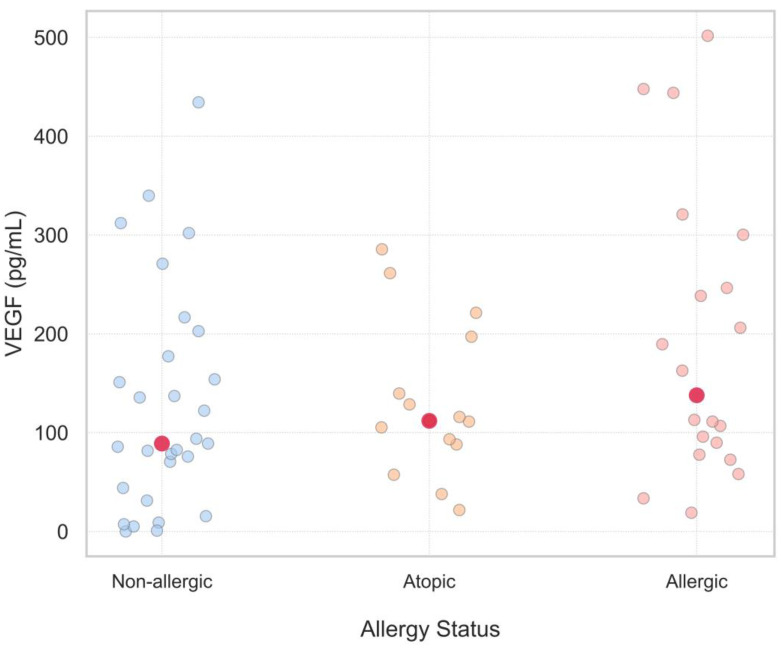
Serum VEGF Levels Across Allergy Status Groups: Non-Allergic, Atopic, and Allergic. This scatter plot shows serum VEGF levels in three groups of allergy status: non-allergic (blue dots), atopic (orange dots), and allergic (pink dots). Each point represents a single measurement. While there is variation within groups, the red dots representing the medians suggest a slight upward trend in VEGF levels with increasing allergy severity. This would indicate a potential association between allergic status and high VEGF levels.

When VEGF values were further compared within 3 subgroups of women: non-allergic, atopic and allergic, serum VEGF levels of infertile women were observed to be higher in all 3 subgroups in comparison with corresponding results of the fertile group; however, the differences were too sophisticated to reach the statistical threshold. In addition, to determine if VEGF levels have the tendency to be higher with the severity of allergic status, a stepwise rise in VEGF levels was noted among the non-allergic, sensitized, and allergic subgroups. The increasing trend was statistically supported by the Jonckheere–Terpstra test (JT = 791.5, *p* = 0.045), suggesting a potential link between VEGF level and allergy status (Figure 2).

**Figure 3:**
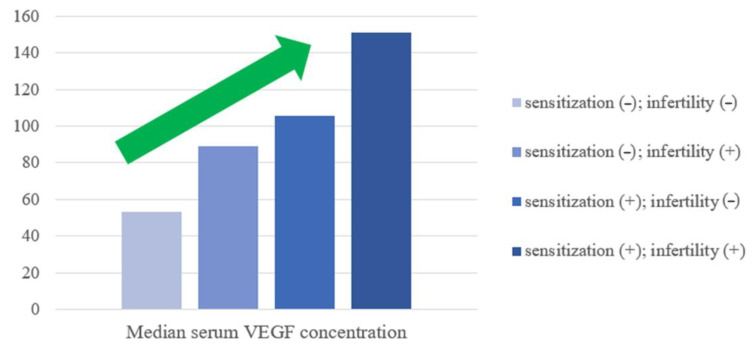
Median serum VEGF concentration variations according to sensitization and fertility status. The green arrow represents the upward trend.

Notably, as higher serum VEGF concentrations were observed for women who were sensitized and for infertile women, the combination of both these features (detected sensitization to at least one allergen component and a diagnosis of unexplained infertility) were established to increase serum VEGF values even more (Figure 3).

According to the ALEX^2^ macroarray test, the most frequently detected sensitizations for all subjects were to pets’, tree pollen and fish and seafood allergen groups: 19 (27.1%) cases, 15 (21.4%) and 15 (21.4%) cases, accordingly. Sensitization to house dust mites (HDM) and grass pollen groups was also frequently detected for all subjects with 14 (20.0%) cases of sensitization for each of these allergen groups. However, the most common allergies (confirmed by a combination of ALEX^2^ test results and clinical history) for all subjects were diagnosed to tree pollen (17 (26.56%)) and cat (16 (25.0%)) allergens (Table 2). Moreover, women with UI were observed to be more frequently sensitized to Fel d 1 (13 (18.6%)) vs. (3 (4.3%)) and Der p 23 (7 (10.0%) vs. 1 (1.43%) in comparison with fertile women of our study.

An interesting observation was discovered when the serum VEGF values were compared between women of all subgroups, who were sensitized to specific allergen groups or components. Firstly, women who were sensitized to pets’ allergens group, were found to have statistically significantly higher serum VEGF concentrations 206.1 pg/mL in comparison with women who were not sensitized to pets’ allergens (93.9 pg/mL) (*p* = 0.003). Secondly, women who were sensitized to weed pollen, were found to have statistically significantly higher serum VEGF concentrations (206.1 pg/mL) in comparison with women who were not sensitized to weed pollen (106.8 pg/mL) (*p* = 0.043).

Women who were sensitized to the cat allergen component Fel d 1 had a significantly higher serum concentration of VEGF, with a median level of 200.0 pg/mL, compared to women who were not sensitized to this allergen. The median level for the non-sensitized group was 100.6 pg/mL, and the difference was statistically significant (*p* = 0.019). Moreover, women, who were sensitized to other pet’s allergens, such as cat’s allergens Fel d 4 (238.4 vs. 111.2 pg/mL), Fel d 7 (238.4 vs. 111.2 pg/mL) and dog’s allergens Can f 1 (214.0 vs. 109.0 pg/mL), Can f 5 (205.4 vs. 109.0 pg/mL), Can f 6 (214.0 vs. 111.2 pg/mL) also had higher median VEGF levels than non-sensitized ones. The highest serum VEGF concentrations of all (501.7 pg/mL and 447.1 pg/mL) were found for women who were both diagnosed with atopic dermatitis and both sensitized to multiple allergen components, of which both were sensitized to: tree pollen (Aln g 1, Bet v 1, Cor a 1.0103, Fag s 1), pets (Can f 1), weed pollen and fruits’ allergen groups.

Additionally, the relationship between serum VEGF concentration and autoimmune diseases in subjects’ clinical history was analyzed. The median of serum VEGF was found to be significantly lower in patients diagnosed with thyroid nodules (N = 3, median VEGF: 15.4 pg/mL) in comparison with patients without thyroid nodules (N = 61, median VEGF: 112.9 pg/mL) (*p* = 0.045). Moreover, though VEGF values were determined to be higher for women with unexplained infertility and women with UI were diagnosed with autoimmune diseases statistically more frequently, according to our study data, no significant tendencies have been observed between levels of serum VEGF concentrations and any other specific chronic or autoimmune disease. Moreover, none of the specific autoimmune, allergic or chronic diseases were diagnosed statistically significantly more frequently for the UI patients in our study.

To establish the range of “normal” serum VEGF concentrations and to facilitate comparison with the data obtained in our study, we analyzed 25 papers published between 1998 and 2024. These papers reported serum VEGF value ranges across various diseases, including oncologic conditions, allergic diseases, as well as chronic, autoimmune, and inflammatory diseases (Table 3).

**Table 3:** The weighted mean VEGF levels in patients vs. controls across 25 publications analyzed.

Disease Group		Sample Size	VEGF Mean (pg/mL)	Reference	Research Types
1.	Oncologic conditions	3401	340.45	[13,14,15,16,17,18,19,20,21,22,23,24,25,26]	Meta-analysis; Prospective cohort observational; Case–control; Observational cohort; Cross-sectional observational
	Breast cancer	1835	216.32	[13,15,16,17,18,19,20]	Meta-analysis; Case–control; Observational cohort; Cross-sectional observational
	Colon cancer	1309	401.83	[13,14,21,22,23]	Meta-analysis; Prospective cohort observational; Observational cohort
	Glioblastoma	90	482.98	[24,25,26]	Case–control
	Other cancers	1005	386.79	[13]	Meta-analysis
2.	Allergic diseases	194	340.45	[10,27,28,29,30]	Pilot observational; Cross-sectional observational; Case–control
3.	Chronic diseases	303	567.90	[31,32,33,34,35,36]	Case–control
4.	Control group	1748	182.68	[10,13,14,15,16,17,18,19,20,21,22,23,24,25,26,27,28,29,30,31,32,33,34,35,36]	Pilot observational; Meta-analysis; Prospective cohort observational; Case–control; Observational cohort; Cross-sectional observational

**Figure 4:**
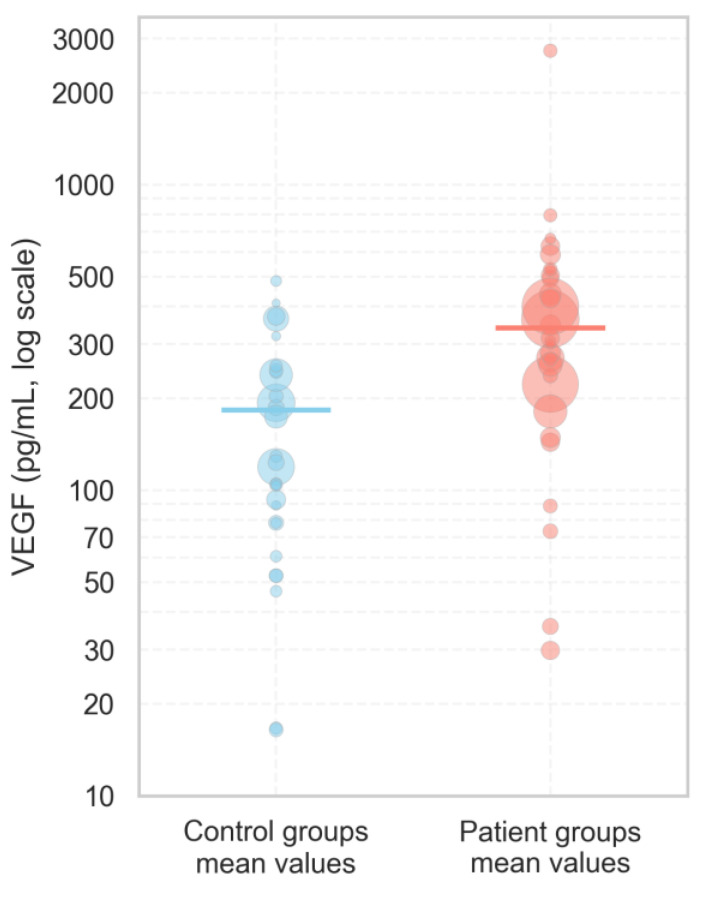
The distribution of serum VEGF concentrations in patients and control groups according to our analyzed literature articles published between 1998 and 2024. Each dot represents the mean VEGF value reported in an individual study, with the size of the dot proportional to the study’s sample size, reflecting the relative weight of each study. Solid horizontal lines indicate the weighted mean VEGF concentration for each group, calculated across all studies.

Based on our thorough scientific analysis of 25 distinct studies, analyzing VEGF concentrations in blood serum, we determined that average VEGF levels in diseased subjects ranged from 29.9 pg/mL to 793 pg/mL, while in healthy controls, the average range was from 16.4 pg/mL to 483.5 pg/mL. The weighted mean VEGF concentration was higher in the diseased patients’ group (338.83 pg/mL) than in the control group (182.68 pg/mL) (Figure 4).

To assess whether the VEGF concentrations observed in fertile women (control group) in our study align with values reported for healthy individuals, we compared our measurements with control data available in the literature. The serum VEGF concentrations in the fertile group (median: 82.5 pg/mL; IQR: 71.7 pg/mL) appeared to be comparable to those reported for healthy controls in published studies (median of reported means: 123 pg/mL; IQR: 164.7 pg/mL). This suggests that the VEGF levels in our control group fall within a physiologically normal range and may be suitable for subsequent statistical analysis and comparison with the infertile patient group.

The most elevated levels of VEGF were measured for patients with oncologic pathologies: in these cases the values of serum VEGF were often double or triple than those seen in healthy controls. Additionally, in a meta-analysis published in 2007 by Kut et al., this finding was also supported by determining that the concentration of VEGF in blood serum was higher in patients with various oncologic diseases [13]. For example, in Kut et al. meta-analysis, serum VEGF level ranges for patients with breast cancer were registered to be about twice as high as those in healthy controls (92–390 vs. 17–287 pg/mL), serum VEGF levels of prostate cancer patients were found to be 2–3 times higher in comparison with healthy controls (129–323 pg/mL for prostate cancer patients vs. 17–171 pg/mL for healthy controls) and serum VEGF levels were found to be approximately twice as high in colorectal cancer patients in comparison with healthy controls (66–563 pg/mL for colorectal cancer patients vs. 173–391 pg/mL for healthy individuals) [13].

In inflammatory diseases, such as bronchial asthma, VEGF levels were also detected to be similarly elevated. For example, in a study by Gomulka et al. VEGF levels were determined to be significantly higher in patients with bronchial asthma in comparison with healthy controls (314.35 pg/mL vs. 246.6 pg/mL, *p* = 0.0131) [27].

## 3. Discussion

In our study, we observed that women diagnosed with unexplained infertility exhibited higher median VEGF levels than fertile controls. Additionally, autoimmune diseases were significantly more frequent in women with UI, though no clear associations were found between individual autoimmune diagnoses and serum VEGF levels. Nevertheless, the highest median serum VEGF concentrations were found for infertile women who were diagnosed with chronic diseases, with the highest serum VEGF level being 501.7 pg/mL.

Elevated serum VEGF levels were also significantly associated with allergic sensitization confirmed by ALEX^2^ macroarray test, with the highest concentrations found in women who were both sensitized (confirmed by ALEX^2^ test) and diagnosed with UI. A statistically significant upward trend in VEGF was observed with increasing allergy severity—rising from non-allergic to atopic, and highest in allergic subjects—pointing towards a possible association between allergy status and elevated level of VEGF. Similar findings were reported by Tedeschi et al. in patients with chronic urticaria, where VEGF levels correlated with disease severity, supporting a link between VEGF and allergy status [37].

Women sensitized to pet allergens—especially to Fel d 1—had significantly elevated VEGF levels. This may indicate that long-lasting exposure to some environmental allergens, leading to continuous immune stimulation, may upregulate VEGF expression. This is supported by studies showing that allergen-stimulated mast cells produce VEGF through leukotriene B4 receptor–2 signaling [38], and that environmental toxicants activating the aryl hydrocarbon receptor (AhR) pathway (e.g., TCDD) enhance VEGF expression in bronchial epithelial cells [39]. IL-9–induced VEGF-A secretion by mast cells also contributes to inflammatory diseases such as atopic dermatitis [40]. Chemical allergens have also been shown to stimulate lymphagiogenic VEGF production by human keratinocytes [41].

Therefore, our results additionally support the hypothesis that elevated serum VEGF level in women with unexplained infertility, particularly those with allergic sensitization, may be a marker for a state of chronic low-grade inflammation or immune dysregulation that interferes with reproductive function. Very recent scientific findings highlight the complex interplay between VEGF, immune system components, and inflammatory processes in the pathophysiology of reproductive disorders [42]. VEGF alone may not serve as a definitive biomarker for infertility; however, substantial evidence links it to fertility outcomes. In IVF patients, VEGF-A concentrations above 43.28 pg/mL are associated with a higher risk of miscarriage or failed embryo transfer compared with lower levels [11]. Altered VEGF expression has also been correlated with reproductive failure, including recurrent implantation failure and recurrent miscarriage [6]. Our results are consistent with those of Atalay et al., who reported significantly elevated VEGF in women with idiopathic recurrent miscarriage compared to healthy fertile women (210.3 ± 108.2 pg/mL vs. 123.9 ± 18.8 pg/mL) [43], supporting VEGF’s potential role as a marker of subclinical inflammation or vascular dysregulation that results in reproductive failure. Importantly, VEGF remains a key molecule in fertility context because of its dual role in immune regulation [44] and angiogenesis [45]—both critical processes for implantation and successful pregnancy. Several studies have demonstrated that VEGF might serve as one of immune modulators and mediate the immuno-tolerance of the maternal immune system during the time of embryo implantation [6]. In UI, immune imbalance is characterized by reduced Treg frequencies, increased Th17 cells, and an elevated Th17/Treg ratio [3] with VEGF implicated in these processes: VEGF-A enhances IFN-γ production, suppresses IL-10, and promotes the polarization of T cells toward a Th1 phenotype [44]. Elevated IL-17 produced by Th17 cells, together with angiogenic cytokines such as VEGF, may drive hypervascularization in endometriosis, facilitating implantation and progression of early lesions [46]. VEGF also acts as a chemoattractant for Tregs [44]. VEGF is linked to Treg cells through the action of G-CSF, which not only expands the Treg population at the fetal–maternal interface but also enhances the pro-angiogenic function of Treg cells, thereby promoting VEGF-mediated trophoblast growth and vascular development [47]. CCL17 and CCL22 recruit Tregs, enhance their immunosuppressive activity, and, together with proinflammatory cytokines, promote angiogenesis in endometrial cells; Tregs contribute to VEGF production in endometriotic lesions by secreting TGF-β1, which, together with IL-1β and TNF-α, activates ERK1/2 and p38 signaling pathways in endometrial stromal cells to promote VEGF secretion and angiogenesis [48]. Tregs further drive angiogenesis in both physiological (pregnancy) and pathological settings (cancer, endometriosis, infertility) through the VEGF/VEGFR axis and by regulating other pro-angiogenic immune cells [49].

One of the main challenges our study has encountered was interpreting VEGF levels and establishing the “normal” serum VEGF value range due to the lack of standardized serum VEGF reference ranges altogether. This currently poses a significant problem for researchers and physicians when evaluating serum VEGF concentration and its significance in different clinical situations. While some equipment manufacturers suggest a reference range of 62–707 pg/mL, there is no universally accepted norm, and VEGF levels are known to vary significantly with pathological conditions [14,50]. Moreover, the medical publications analyzing only female VEGF levels are scarce.

Similarly, in the context of assisted reproduction technologies (ART), specific VEGF levels have been associated with both, ovarian hyperstimulation syndrome (OHSS), which is one of the most common complications of ART, as well as implantation success itself. However, while elevated VEGF levels are not always necessarily pathogenic, as in cases of OHSS, elevation of this biomarker may be indicative of systemic inflammation or vascular reactivity [51]. Our results support the thesis that increased levels of VEGF may reflect an inflammatory state influenced by associated conditions such as atopy or chronic illness, which may be significant for the impaired reproductive function.

In allergic diseases, VEGF contributes to Th2-type inflammation, promoting asthma-like phenotypes [52], recurrent wheezing [53], and nasal obstruction and inflammation in response to allergens [54]. In chronic diseases, VEGF is upregulated in response to hypoxia via HIF-1α–driven signaling, leading to angiogenesis and increased vascular permeability [55,56]. In oncologic conditions, serum VEGF levels also increase mainly due to hypoxia-induced signaling and tumor-driven angiogenesis. Rapid tumor growth causes hypoxia, which activates HIF-1 and upregulates VEGF to support vascularization and metastasis [57]. Oxidative stress under hypoxic conditions stabilizes HIF-1α, further enhancing VEGF expression through the NF-κB signaling pathway, which also promotes inflammation, tumor progression and angiogenesis [58,59]. Cytokines such as TGF-β1, IL-1β, IL-4, and IL-13 promote VEGF production, contributing to neovascularization and inflammation in conditions like allergic conjunctivitis and asthma [41,60]. Additionally, PI3K/Akt pathway is crucial in regulating VEGF expression. In vivo studies show that VEGF inhibition reduces TGF-β1 levels and fibrosis, indicating a feedback loop between VEGF and TGF-β1 via PI3K/Akt signaling [61]. VEGF-C also contributes to lymphangiogenesis in skin sensitization [41]. Elevated VEGF levels, which were observed for our subjects sensitized to pets, and particularly cats, further support the notion that persistent antigen exposure in chronic sensitization processes—such as unavoidable pet-related sensitization—may contribute to increased VEGF concentrations.

To sum up the above mentioned complex pathophysiological pathways in each of which VEGF plays a crucial role, the absence of clear norms makes it difficult to determine what constitutes “normal” VEGF level range, especially in conditions as varied as infertility, cancer, and allergic diseases, where VEGF expression may be affected by a complex of underlying factors [14,50].

From a reproductive perspective, based on its mode of action, VEGF is a key mediator of increased vascular permeability, negatively affecting oocyte maturation and follicular development in cases of OHSS [62,63,64]. Additionally, elevated VEGF levels in follicular fluid has been correlated with lowered ovarian reserve and oocyte maturation rates. This is supported by a negative correlation between VEGF levels and the number of oocytes retrieved, as well as peak estradiol levels in the studies published by Wu et al. These factors are crucial for successful fertilization and embryo development [63,65].

However, other studies (e.g., Monteleone et al.) suggest positive associations of higher VEGF levels with better perifollicular perfusion, which can lead to improved oocyte fertilization rates and embryo quality [66]. However, imbalanced VEGF levels may impair endometrial receptivity and embryo implantation—processes in which VEGF also play an important role—thereby contributing to implantation failure [67]. Friedman et al. and Asimakopoulos et al. reported that elevated VEGF levels in follicular fluid and blood serum, respectively, were associated with lower IVF success rates [65,68].

Overall, VEGF is a critical inflammatory biomarker involved in key reproductive processes, including oocyte maturation, endometrial receptivity, and endometrial remodeling and regeneration during each menstrual cycle [69]. Both over- and underexpression can have negative reproductive consequences.

Nevertheless, given VEGF’s involvement in chronic diseases, allergies, and cancer, elevated VEGF levels may not directly cause female infertility but rather reflect a complex interplay of coexisting pathologies that indirectly affect reproductive function. VEGF could also serve as a biomarker of underlying processes such as oxidative stress, which may be influenced by conditions like endometriosis or hypoxia linked to allergies and chronic disease [27,53,54,70,71]. These facts underscore the undeniable need for further studies to elucidate the mechanistic effects VEGF action and its associations with other parameters in the context of infertility. Because infertility is a multifactorial condition, it is likely that no single biomarker can fully address its complexity; therefore, VEGF should also be investigated in combination with other parameters to better clarify its role. However, the current literature on the use of VEGF in combination with broader biomarker panels for the diagnosis or prediction of infertility remains limited, highlighting the need for integrative approaches that consider multiple immunological and inflammatory mediators. In 2023, the combination of CXCL-6 and VEGF has been shown to better predict oocyte maturity in IVF by Chen HT et al. [72]. Although we identified significant associations between VEGF and allergy status, the Alex microarray assay integration with the VEGF concentrations did not yield higher discriminatory power regarding fertility status. In addition, a limitation of the study was the relatively small sample size, particularly in the control group, which highlights the need for future research with larger, well-characterized cohorts to clarify VEGF’s role in female reproductive health, evaluate the impact of comorbidities, and assess potential gender-related differences. The absence of female-specific VEGF reference values presents a diagnostic challenge, highlighting the need for gender-dependent normative data.

## 4. Materials and Methods

A prospective observational study was conducted. A total of 70 patients, who were referred to the Centre of Innovative Allergology in Vilnius, Lithuania were included in the study: 51 women with unexplained primary infertility and 19 fertile women. The inclusion and exclusion criteria are described in Table 4.

Vilnius Regional Biomedical Research Ethics Committee reviewed and approved the implementation of this study (document number 2024/2-1558-1026, approved on 22 January 2025). Individuals who agreed to participate in this study signed written informed consent forms.

The dataset consisted of the demographic data of the subjects, ALEX^2^ macroarray test results, concentrations of vascular endothelial growth factor (VEGF) in blood serum determined by the enzyme-linked immune sorbent assay (ELISA) method as well as gynecological and allergological–immunological anamnesis of the participants. Gynecological and allergological–immunological anamnesis included information about previously diagnosed pathologies, which could have an impact on female fertility, as well as the results of previously performed tests to diagnose gynecological and allergological–immunological pathologies. Venous blood for ALEX^2^ and ELISA tests was drawn via venipuncture into Vacutainer tubes without anticoagulant for serum collection. The blood was left to clot at room temperature, centrifuged, and the separated serum was frozen at −80 °C.

Serum VEGF concentration was measured in duplicate using a commercial ELISA kit from Thermo Fisher Scientific. The assay had a sensitivity of less than 5 pg/mL of VEGF. Optical density at 450 nm was measured using a BioTek ELx800 plate reader (Winooski, VT, USA). VEGF concentrations were determined by linear regression from a standard curve generated with the VEGF standards provided in the kit.

The sensitization of the subjects was analyzed using the ALEX^2^ macroarray test (MacroArray Diagnostics GmbH, Austria, Vienna), an ELISA-based immunoassay. The ALEX^2^ test includes 295 antigens (117 extract allergens and 178 molecular components) bound to nanoparticles and arrayed on a solid surface. In the assay, particle-bound allergens interact with specific IgE antibodies in the patient’s serum sample. After incubation, nonspecific IgE is removed by washing. An enzyme-labeled anti-human IgE detection antibody is then added, forming complexes with the particle-bound specific IgE. After a second wash, a substrate is introduced, producing an insoluble colored precipitate. The reaction is stopped with a blocking reagent, and the precipitate amount correlates with the specific IgE concentration. Image acquisition and analysis were performed using the ImageXplorer device, and results were processed with MADx’s RAPTOR SERVER Analysis Software v1.18. IgE levels were reported in kUA/L, with total IgE in kU/L. Sensitization was defined by an IgE level of 0.3 kUA/L or higher.

According to ALEX^2^ macroarray test results and their clinical history, all patients were additionally allocated into three subgroups by “allergy status”: non-allergic (negative ALEX^2^ macroarray test results), atopic (positive ALEX^2^ macroarray test results without clinical history of allergy to respective allergens) and allergic (positive ALEX^2^ macroarray test results and previous clinical history of allergy to respective allergens).

Scientific literature review of articles published between 1998 and 2024 reporting serum VEGF value ranges was performed in order to establish the range of “normal” serum VEGF concentrations for healthy individuals and to facilitate comparison with the data obtained in our study. The articles were only included in the review if they reported VEGF values in of the blood serum and if they featured a comparison of VEGF values between healthy and diseased individuals. In addition, we also analyzed reports describing possible combinations or panels of VEGF with other biomarkers relevant to infertility in order to contextualize our findings within broader immunological and inflammatory networks.

The Shapiro–Wilk test was utilized to evaluate the normality of the data. Pairwise comparisons were performed using the Mann–Whitney U test with a two-tailed hypothesis and the Kruskal–Wallis test was applied to assess differences between patient groups. The Jonckheere–Terpstra test was employed to evaluate ordered differences among multiple groups. Categorical data variables were compared using Fisher’s Exact test. Logistic regression combined with ROC curves was used to evaluate the classification performance of various models. The optimal cut-off value for serum VEGF concentration was determined using Youden’s J statistic. All analyses were conducted using the R program (version 4.3.3) with the Rcmdr package (version 2.9-2), and Python version 3.11.4 (Python Software Foundation), with statistical significance defined as *p* < 0.05.

## 5. Conclusions

Our study determined that women with UI, particularly those with allergic sensitization, had elevated serum VEGF levels in comparison with fertile women. Sensitization to pet allergens—especially cat allergen Fel d 1—was associated with the highest VEGF concentrations, and VEGF levels increased progressively with allergy status: non-allergic > atopic > allergic. Additionally, women with UI were more frequently diagnosed with autoimmune diseases. These findings suggest that VEGF may reflect broader immunological dysregulation relevant to fertility.

Literature analysis confirmed substantial variability in serum VEGF levels between healthy individuals and those with oncological or inflammatory diseases. Importantly, the VEGF values observed in our control group aligned with those reported for control groups in the literature, suggesting that our defined “normal” range may be appropriate for subsequent statistical analysis.

Further research is essential to determine whether elevated serum VEGF levels could directly contribute to specific infertility-related mechanisms or whether detected VEGF levels merely reflect an individual’s overall disease burden, with chronic conditions or allergic sensitization and their associated inflammatory environment being the primary contributors to reduced fertility. If VEGF is confirmed to be directly involved in infertility pathogenesis, it could serve as a valuable biomarker for identifying underlying causes of infertility that are currently categorized as unexplained. However, even if VEGF is not found to have a direct effect on female fertility or to reliably pinpoint a specific infertility-causing condition in future in-depth research, its elevated levels may still indicate a heightened inflammatory or immune-activated state that could adversely affect reproductive function given the critical role of well-balanced VEGF levels for optimal fertility out-comes.

Given its association with both allergic sensitization and unexplained infertility, VEGF testing in women with UI—especially in the presence of atopy—can already serve as a useful tool for prompting broader clinical evaluation. It can help identify coexisting chronic, autoimmune, or inflammatory conditions that are potentially contributing to reduced fertility. Even if not directly causal, elevated VEGF reflects a physiologically unfavorable environment for conception, thereby highlighting women who could benefit from targeted diagnostic work-up and potentially more personalized management strategies. Moreover, the analysis of not only a single biomarker such as VEGF but also a broader panel of biomarkers is crucial, as it may better capture the complex immunological and inflammatory networks underlying unexplained infertility. Further in-depth research is essential to determine the potential direct role of VEGF in infertility.

## Figures and Tables

**Table 1:** Distribution of VEGF concentration in blood serum.

Groups	VEGF Median (pg/mL)	Q1 (pg/mL)	Q3 (pg/mL)	IQR (pg/mL)	*p* Value
Fertile women	82.5	57.8	128.9	71.1	0.152
Women with unexplained infertility	128.6	80.0	219.0	139.0	
All sensitized women	115.9	89.8	238.4	148.6	0.028
All non-sensitized women	85.7	21.7	177.3	155.6	
Sensitized fertile women	105.4	75.2	150.7	75.6	0.287
Non-sensitized fertile women	53.5	18.5	125.6	302.0	
Sensitized women with unexplained infertility	151.2	98.7	244.5	145.8	
Non-sensitized women with unexplained infertility	89.0	44.0	197.1	153.1	
Atopic fertile women	108.3	69.4	111.8	42.4	0.376
Atopic women with unexplained infertility	128.6	93.3	197.1	103.8	
Allergic fertile women	77.7	72.6	189.5	116.9	
Non-allergic fertile women	79.2	25.7	125.6	99.9	
Allergic women with unexplained infertility	162.7	101.4	273.4	172.0	
Non-allergic women with unexplained infertility	93.9	70.6	202.7	132.1	

**Table 2:** The most common sensitizations and diagnosed allergies among all study participants.

Allergen Group/Allergen	All Subjects	Fertile Women	Infertile Women
**Sensitizations (positive ALEX^2^ test result)**			
Pet allergens	19 (27.1%)	4 (5.7%)	15 (21.4%)
Tree pollen	15 (21.4%)	5 (7.1%)	10 (14.3%)
Fish/seafood	15 (21.4%)	5 (7.1%)	10 (14.3%)
House dust mites (HDM)	14 (20.0%)	3 (4.3%)	11 (15.7%)
Grass pollen	14 (20.0%)	3 (4.3%)	11 (15.7%)
**Confirmed Allergies (positive ALEX^2^ test result and clinical history)**			
Tree pollen	17 (26.6%)	4 (6.3%)	13 (20.31%)
Cat allergen	16 (25.0%)	4 (6.3%)	12 (18.8%)

**Table 4:** Description of inclusion and exclusion criteria.

Category	Infertile Group	Control Group
Inclusion criteria	Consent to participate	Consent to participate
	Female sex, 18–43 years old	Female sex, 18–43 years old
	No clinical signs of menopause	No clinical signs of menopause
	No current pregnancy or breastfeeding	No current pregnancy or breastfeeding
	No oncologic conditions (current or previous)	No oncologic conditions (current or previous)
	Primary infertility: no previously confirmed pregnancies	Fertility proven: at least one verified pregnancy in the clinical history
	Unexplained infertility: diagnosis established after excluding anatomical, hormonal, infectious, and other causes of infertility	No clinical history of infertility
Exclusion criteria	Established causes of infertility confirmed: male-factor infertility, anatomic abnormalities or physical blockage of female reproductive tract etc.	Severe comorbidities impairing health/reproductive function
	Vulnerable individuals *	Vulnerable individuals *
		Confirmed chronic diseases, which are not controlled or in remission

* People who, due to their health condition, cannot be regarded as capable of rationally assessing their interests; children; students, if their participation in biomedical research is related to studies; people living in social care institutions; soldiers during their actual military service; health care institutions where a biomedical examination is carried out, employees subordinate to the researcher; people in prisons or other places of deprivation of liberty.

## Data Availability

Data is contained within the article.
